# The Increasing Prevalence of Autism Spectrum Disorder in the U.S. and Its Implications for Pediatric Micronutrient Status: A Narrative Review of Case Reports and Series

**DOI:** 10.3390/nu17060990

**Published:** 2025-03-12

**Authors:** Kamsiyochukwu S. Daniel, Qianzhi Jiang, Margaret S. Wood

**Affiliations:** 1 Texas Children’s Hospital, Houston, TX 77030, USA; 2 Jiang Nutrition LLC, Natick, MA 01760, USA; 3 Nutrition and Health Studies Department, Framingham State University, Framingham, MA 01701, USA; 4 Marlboro Institute of Liberal Arts and Interdisciplinary Studies, Emerson College, Boston, MA 02116, USA

**Keywords:** autism spectrum disorder, micronutrients, nutritional deficiencies, vitamin A deficiency, restrictive eating, food selectivity

## Abstract

Introduction: Micronutrient deficiencies are considered uncommon in the United States. However, children with autism spectrum disorder (ASD) are at higher risk due to food selectivity and restrictive eating patterns. The prevalence of ASD in the U.S. has quadrupled over the past two decades, amplifying the need to address nutritional gaps in this population. Objective: This narrative review examines the prevalence and clinical impact of underreported micronutrient deficiencies beyond vitamin C in children with ASD using case reports and series. Methods: Case reports and case series reporting micronutrient deficiencies in children with ASD published from 2014 to 2025 were identified through PubMed and ScienceDirect using search terms “autism and deficiency” and “autism and vitamin A, K, magnesium, iron deficiency”. Eligible cases included children aged 2–18 years with ASD and laboratory-confirmed micronutrient deficiencies. Results: A total of 44 cases from 27 articles were analyzed. Frequently reported deficiencies were vitamin D (25.0%), vitamin A (24.8%), B-vitamins (18.0%), calcium (10.8%), and iron (9.6%). Less common deficiencies included iodine, zinc, vitamin E, etc. Diseases such as xerophthalmia, rickets, pellagra, and goiter were reported. Co-occurring deficiencies were present in 70% of cases, and all cases reported food selectivity, with deficiencies occurring despite normal growth parameters in some children. Conclusions: Based on cases reviewed, children with ASD are at high risk for micronutrient deficiencies, despite meeting normal growth parameters. Further research is needed to develop a standardized nutrition assessment, but combining anthropometric, biochemical, and dietary assessments can aid in early intervention and prevent complications.

## 1. Introduction

Micronutrients are essential for supporting optimal growth, immune function, and cognitive development during childhood. Imbalances in micronutrient intake—deficiencies or excesses—can have health consequences, including stunted growth, increased susceptibility to infections, impaired neurodevelopment, or toxicity [1,2,3]. While the rate of micronutrient deficiencies per biochemical assessment has been reported to be between 1 and 10% [4], and toxicities are rare in the United States [5], children with autism spectrum disorder (ASD) represent a uniquely vulnerable population. Autism Spectrum Disorder is a complex neurodevelopmental disorder often characterized by impairments in social interactions and repetitive and/or restrictive behaviors, with onset in early childhood as defined by the Diagnostic and Statistical Manual of Mental Disorders 5th edition (DSM-5) by the American Psychiatric Association [6]. These behavioral traits often extend to nutritional difficulties, beyond macronutrients and physical growth.

Dietary challenges commonly associated with ASD—including selective eating behaviors, sensory sensitivities, and rigid food preferences for specific textures, colors, tastes, appearances, and smells—often lead to restrictive dietary patterns [7,8,9,10]. These challenges have been reported to affect up to 89% of children with ASD, making them more significantly prone to nutritional imbalances compared to neurotypical peers [7]. Selective eating in ASD often goes beyond the typical picky eating observed in young children. Many individuals exhibit extreme food aversions, consuming only a limited number of accepted foods while rejecting entire food groups. This can be due to sensory hypersensitivities, where certain textures (e.g., mushy, crunchy, or fibrous foods) trigger strong negative reactions, including gagging, vomiting, or refusal to eat, particularly with fruits and vegetables, with a preference for processed and carbohydrate-rich foods which are more calorie dense [8,10]. These rigid food preferences limit dietary diversity, particularly in micronutrients, as nutrient-dense foods are more likely to be rejected. The underlying mechanism or etiology of food selectivity within this population is not fully understood.

The prevalence of ASD in the U.S. has increased dramatically over the past two decades. According to the Autism and Developmental Disabilities Monitoring (ADDM) Network, ASD rates rose from 0.7% in 2000 to 2.8% in 2020—a fourfold increase, with notable disparities between boys (4%) and girls (1%) [11]. While improved screening and awareness may contribute to this trend, biological and environmental factors may also play a role [12,13]. Nevertheless, as the prevalence of ASD continues to rise, so does the need to address unique nutritional challenges associated with the condition.

Although anthropometric measurements are crucial for evaluating nutritional status, research has demonstrated that children with ASD and normal growth velocity may still experience micronutrient deficiencies [14], highlighting a need for comprehensive nutritional assessment beyond physical growth. While vitamin C deficiency has been well-documented in children with ASD, it is excluded from this review due to its frequent coverage in the existing literature and its relatively well-established clinical presentation, such as through scurvy [14,15]. Deficiencies in micronutrients such as vitamin A, B vitamins, and iodine, less documented in the literature, may receive comparatively less clinical attention, increasing the risk for underdiagnosis and suboptimal management.

To our knowledge, only two reviews have examined micronutrient deficiencies in similar U.S. populations using case reports [14,15]. However, one included approximately 60% international cases, while the other focused primarily on vitamin C deficiency, and both incorporated cases predating the adoption of DSM-5 diagnostic criteria. This narrative review examines the prevalence and clinical impact of underreported micronutrient deficiencies in children with ASD beyond vitamin C, using previously published case reports and series. The information presented in this review will highlight the importance of micronutrient assessments, including biochemical assessments, as there are no routine laboratory measures for nutritional adequacy after 12 months of age recommended by the American Academy of Pediatrics (AAP) [16].

## 2. Methods

### 2.1. Search Strategy and Data Source

The search was conducted by two authors for articles published from 2014 to January 2025, on the PubMed and ScienceDirect electronic databases. Case reports and series were identified through databases and by scanning bibliographies of identified articles. We used a combination of controlled vocabulary (e.g., MeSH terms) and free-text keywords related to ASD and micronutrient deficiencies. The search terms included the following: Population Terms: “autism spectrum disorder”, “ASD”, “autistic children”, “pediatric autism”. Deficiency Terms: “micronutrient deficiency”, “vitamin deficiency”, “mineral deficiency”, “nutritional deficiency” Specific Nutrients: “vitamin D”, “vitamin A”, “iron”, “calcium”, “iodine”, “vitamin B12”. Additional Terms: “dietary intake”, “food selectivity”, “picky eating”, “restrictive eating”. Search String Example: (“autism spectrum disorder” OR “ASD” OR “autistic children”) AND (“micronutrient deficiency” OR “vitamin deficiency” OR “mineral deficiency” OR “nutritional deficiency” OR “vitamin D” OR “vitamin A” OR “iron” OR “calcium” OR “iodine” OR “B vitamins”) AND (“dietary intake” OR “food selectivity” OR “picky eating” OR “restrictive eating”).

The initial search was limited to the title, abstract, and key words used. Abstracts and citations of all retrieved articles were imported into Zotero (7.0.13), duplicates were removed, and articles were screened for eligibility. Initially, a search was conducted to find cases of deficiencies and toxicity; however, no results were found for excessive micronutrient intake or toxicity, thus leading to our focus on deficiencies.

### 2.2. Inclusion and Exclusion Criteria

This review included original cases reports, as well as reports documenting retrospective chart reviews of previously published case reports and case series in which children from ages 2 to 18 years old were diagnosed with autism spectrum disorder prior to or during a clinical encounter; a biochemical analysis was performed to assess nutritional deficiency and reports of self-imposed food selectivity/restriction. Reports in which a case was deficient in nutrients but received no diagnosis of a nutrition deficiency disease were included. For example, it is possible to have inadequate levels of calcium without the diagnosis of hypocalcemia. It was important to include such cases, as the overall goal for this review is to highlight the importance of assessing micronutrient status in this population to prevent severe deficiency. Articles were limited to the past 10 years and to the United States. While case reports prior to 2014 were not included in this review, we acknowledge there may have been some reported cases of micronutrient deficiencies predating DSM-5. However, our focus was on more recent studies that reflect current diagnostic standards of ASD.

**Figure 1:**
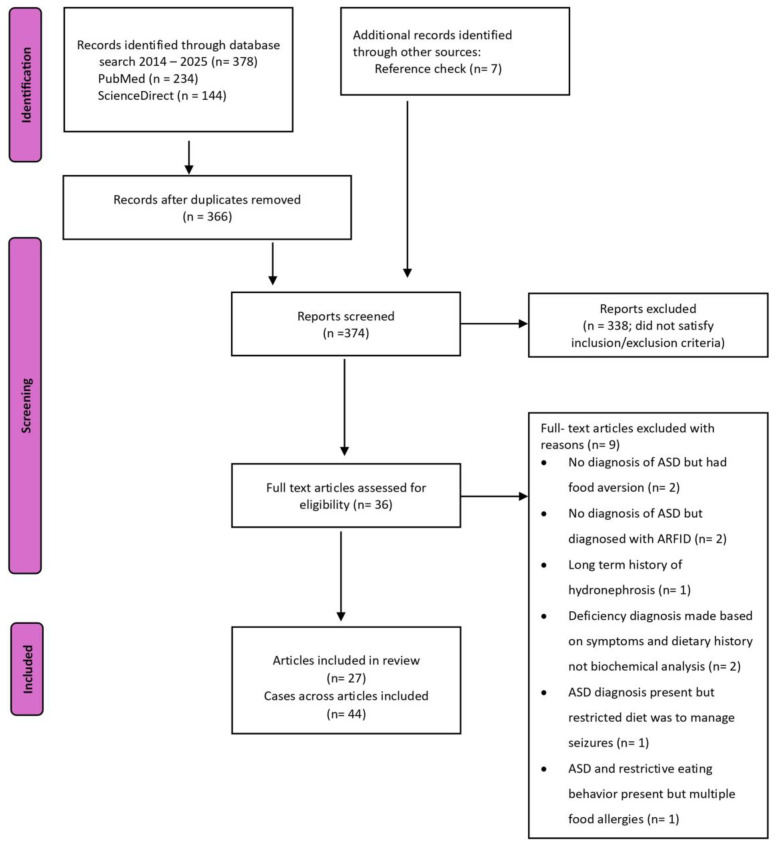
Preferred Reporting System Items for Systematic Reviews and Meta-Analyses [18] flow diagram of the literature search and filtering results for a review of micronutrient deficiencies in children with autism spectrum disorder (ASD) using case reports and series. ARFID = Avoidant Restrictive Food Intake Disorder.

Cases in which the individual had a pre-existing medical condition such as cancer, food allergies, cystic fibrosis, gastrointestinal disorders (Crohn’s or ulcerative colitis), eating disorder diagnosis (anorexia nervosa, bulimia nervosa, and binge-eating disorder), or extreme poverty and neglect were excluded. Cases reporting only vitamin C deficiency were excluded; however, vitamin C deficiency reported concurrently with other vitamin/mineral deficiencies was included. Cases reporting the long term use of medications with drug-nutrient interactions such as corticosteroids, proton pump inhibitors, diuretics, etc. [17], were excluded. A total of 44 cases from 27 articles were used in this review. Although not a systematic review, authors adhered closely to the PRISMA 2020 guideline presented in the flow diagram (Figure 1) [18].

## 3. Results

**Figure 2:**
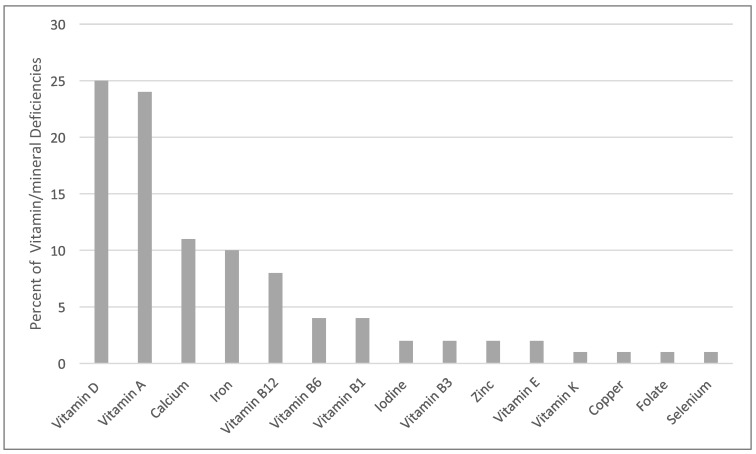
Percentage of vitamin and mineral deficiencies from a review of case reports and series on micronutrient deficiencies in children with autism spectrum disorder (ASD).

**Figure 3:**
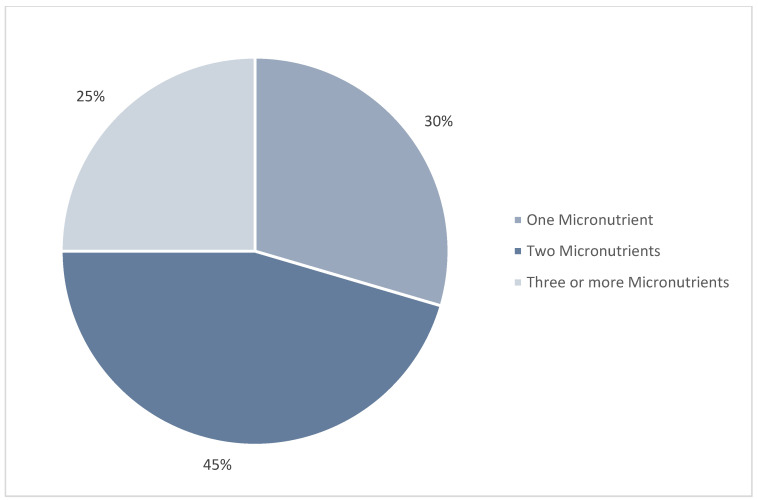
Occurrence of single or multiple micronutrient deficiencies from a review of case reports and series on micronutrient deficiencies in children with autism spectrum disorder (ASD).

This review analyzed 44 reported cases of micronutrient deficiencies in pediatric patients with ASD in the U.S. over the past decade (Table 1 & Supplementary Material Table S1) [19,20,21,22,23,24,25,26,27,28,29,30,31,32,33,34,35,36,37,38,39,40,41,42,43,44,45]. The most frequently reported deficiencies were vitamin D (25.0%), vitamin A (24.1%), B-vitamins (18%), calcium (10.8%), iron (9.6%), and iodine (2.4%) (Figure 2). Many cases exhibited multiple micronutrient deficiencies beyond the primary nutrients associated with symptoms (Table 1, Figure 3). Dietary patterns were consistently described as restrictive or selective, with terms such as “picky eating”, “restrictive eating”, and “food selectivity”. Although anthropometric data were not reported in all cases, 59% included some form of measurement. Among these, 38.5% included only weight in kilograms or percentiles, and/or comments on weight velocity and body size such as “thin body habitus”, while 61.5% provided BMI data or sufficient information for calculation. Among cases with BMI data, 50% fell within the normal range (5th–84th percentile), while the remaining 50% met criteria for overweight or obesity (≥85th percentile) based on the CDC growth chart for 2–20 years [46]. Notably, no cases met the criteria for underweight (<5th percentile).

### 3.1. Vitamin D and Calcium

Vitamin D was identified as the most common deficiency among this population (Figure 2), often accompanied by calcium deficiency; all cases of hypocalcemia also had inadequate vitamin D (Table 1). Vitamin D and calcium are critical for bone health during childhood, with deficiencies potentially resulting in weakening and softening of the bones, leading to conditions such as rickets and osteomalacia. Vitamin D is obtained through dietary sources including fatty fish, fortified foods, and supplements, and synthesized in the skin via sunlight exposure. However, factors such as season, time of day, weather, skin pigmentation, and sunscreen use can limit vitamin D synthesis. In the U.S., fortified dairy products are the primary dietary sources of vitamin D [47]. Notably, dairy was absent in the dietary history of many reported cases in this population, though some included cheese, which is typically not fortified. For instance, while one serving of 2% milk (8 ounces) contains approximately 120 international units (IU) of vitamin D, a serving of cheddar cheese (1.5 ounces) provides only 17 IU [48].

The frequent testing for vitamin D in the U.S. compared to other micronutrients may have contributed to its prominence in the findings of this review. Consequently, while vitamin D deficiency is prevalent in the general U.S. population, with only 34.5% meeting sufficient levels [49], rickets remains rare but appears to be reemerging in certain vulnerable populations. The prevalence of rickets in the U.S. increased from 3.7 per 100,000 in 1990 to 24.1 per 100,000 in 2000 [50]. Yule et al. documented three cases of rickets in children with ASD in 1993, 2018, and 2019 in the U.S. [14]. We report five cases of rickets [23,30,38,39,41] and three cases of osteopenia (low bone mineral density) [31,34,45] associated with vitamin D deficiency and/or hypocalcemia. Notably, two cases required orthopedic surgery due to severe complications, including a displaced fracture [26] and genu valgum (knock knee) [30]. Overall, 21 cases of vitamin D deficiency and 9 cases of calcium deficiency were identified, with some presenting ambulatory challenges but not progressing to rickets or osteopenia. Timely diagnosis and intervention can potentially prevent or delay symptoms and the necessity of orthopedic surgery.

### 3.2. Vitamin A

Vitamin A deficiency is uncommon in the United States (<1%) [1]; however, this review identified it as the second most frequently reported deficiency, often leading to xeropthalmia (Table 1, Figure 2). Beyond its role in vision, vitamin A is critical for immune function and deficiency is associated with increased susceptibility to infections due to its role in maintaining mucosal barriers [51]. Thus, children with ASD may be at greater risk not only for ocular complications but also for weakened immune response.

As a fat-soluble vitamin stored in the liver, vitamin A deficiency can take years to manifest. Xeropthalmia (spectrum of ocular disease) is the most common clinical presentation, occurring after prolonged depletion of the body’s vitamin A reserves [51]. Signs and symptoms include nyctalopia (night blindness), keratomalacia (corneal softening), and conjunctival xerosis (dry conjunctiva). Thus, in most cases, a long history of inadequate intake precedes these manifestations. Dietary sources include liver, fish, eggs, fortified dairy products, leafy greens, sweet potatoes, fortified cereal, etc. [51], most of which were absent in the cases presented. We identified 20 cases of vitamin A deficiency, 13 of which led to xeropthalmia, and most resolved with vitamin A supplementation (Table 1); however, 2 cases resulted in irreversible blindness [21]. Similar cases of xerophthalmia in ASD patients with limited dietary patterns have been reported in other developed countries, including Canada [52], Japan [53,54], Ireland [55] and Australia [56]. In addition to xeropthalmia, specific symptoms such as keratomalacia and nyctalopia were commonly reported.

### 3.3. B-Vitamins

B-vitamins are a group of water-soluble vitamins essential for key physiological functions, including energy metabolism, DNA synthesis, red blood cell production, and neurological health. This group comprises vitamins B1 (thiamin), B2 (riboflavin), B3 (niacin), B5 (pantothenic acid), B6 (pyridoxine), B7 (biotin), B9 (folate), and B12 (cobalamin), all of which play critical roles in enzyme function and maintaining nervous system integrity [57].

Among the B-vitamins, vitamin B12 deficiency was the most frequently reported, followed by deficiencies in B6, B1, and B3 (Figure 2). The body stores approximately 1 to 5 mg of cobalamin—1000 to 2000 times the average daily intake—and thus clinical symptoms of deficiency can take years to manifest [58]. Consequently, vitamin B12 deficiency was not typically identified as a primary deficiency but was often secondary to deficiencies in other nutrients, such as vitamin A [21,28]. Nevertheless, symptoms such as optic neuropathy (progressive vision loss) was reported in one case [21]. Plant-based foods do not naturally provide this vitamin; fortified breakfast cereals, nutritional yeasts, and animal-derived foods such as meat and dairy products are primary dietary sources [58]. Cases of B12 deficiency in this population revealed dietary patterns heavily reliant on processed foods such as chicken nuggets, crackers, chips, pizza, bread sticks, waffles, etc. (Table 1). Unlike vitamin B1, B3, and folate, B12 is not a mandatory fortification requirement for foods labeled as enriched flour in the U.S. according to the Food and Drug Administration (FDA) regulation (21 CFR 137.165) [59]. This may have contributed to its higher prevalence compared to other B-vitamin deficiencies due to its absence in enriched flour.

Similar to B12, deficiencies in B1 and B6 were secondary to other nutrient deficiencies (Table 1), with three cases requiring enteral nutrition support (nasogastric or gastric tube) due to severe restrictive dietary pattern [25,44]. However, B1 deficiency, while rare in the U.S. [60], was reported in two severe cases, leading to pellagra causing photosensitive dermatitis [22,36]. Neither case was associated with other vitamin or mineral deficiency and was resolved within days following niacin supplementation. The dietary patterns of these cases notably excluded grains and fortified cereals (Table 1), which are primary sources of niacin in the U.S. [60].

### 3.4. Iron

Iron deficiency remains the most prevalent single-nutrient deficiency among children in the United States, with a reported prevalence of 7.1% in children aged 1–5 years as of 2007–2010 [61]. Unlike other micronutrients, hemoglobin levels are routinely assessed as part of a complete blood count (CBC), allowing for the earlier detection of iron-related issues. However, preventative measures remain critical, as ferritin is a more sensitive marker for the early detection of iron deficiency even when hemoglobin levels are within normal range [62].

Yule et al. [14] identified chicken nuggets as the most commonly consumed meat product in children with ASD, which aligns with the dietary patterns observed in this review. Among cases reporting iron deficiency, diets were often characterized by the consumption of foods such as chicken nuggets, French fries, pizza, bread, cheese crackers, and cereal, with limited intake of iron-rich foods (Table 1). Although cereals are frequently fortified with iron, the amount varies widely by brand, and young children may not consume sufficient quantities to meet dietary needs [63]. While some cases reported cereal consumption, details regarding portion size, frequency, and overall intake remain unclear, further complicating the evaluation of dietary adequacy. Additionally, the AAP notes that 60% of anemia in children is not attributable to iron deficiency, and most toddlers with iron deficiency do not present with anemia [63]. Consistent with this observation, only one of the eight cases of iron deficiency in this review reported a diagnosis of anemia, suggesting that routine hemoglobin assessment may help identify iron deficiency anemia. However, ferritin assessment may identify iron deficiency before it progresses to anemia. Notably, iron deficiency in these cases was frequently accompanied by vitamin C deficiency, which plays a key role in enhancing non-heme iron absorption [1], thus underscoring the importance of assessing coexisting nutrient deficiencies when managing iron deficiency in this population.

### 3.5. Iodine

Iodine is essential for the synthesis of thyroid hormones triiodothyronine (T3) and thyroxine (T4), which regulate critical biological processes such as metabolism, skeletal development, and neurological maturation in fetuses and infants. As a result, adequate iodine intake is particularly important during pregnancy and early childhood [64]. Insufficient iodine intake can lead to hypothyroidism, often accompanied by goiter, which is typically the earliest clinical manifestation of deficiency. In the U.S., dietary iodine is primarily obtained from dairy products, iodized salt, and bread, while seaweed and seafood—the richest sources of iodine—are less commonly consumed [65].

The National Health and Nutrition Examination Survey (NHANES) data from 2001 to 2020 indicate iodine sufficiency among children aged 6–11 years, and deficiency is considered uncommon in the general population due to the introduction of salt iodization in the 1920s [66]. This review identified two cases of iodine deficiency leading to clinically apparent goiter in children with ASD (Table 1); both cases reported no dairy and seafood consumption [24,42]. In each case, goiter and abnormal thyroid-stimulating hormone (TSH) and T4 levels improved with iodine supplementation; however, the resolution of goiter required substantial recovery time. Though iodine deficiency is rare in the U.S., children with ASD may face an elevated risk due to restrictive eating behaviors, highlighting the need for heightened awareness and early nutritional intervention to safeguard cognitive health.

### 3.6. Other Micronutrients of Concern

Although vitamin D, vitamin A, calcium, iron, and B-vitamins were the most reported deficiencies, other micronutrients such as zinc, vitamin E, vitamin K, folate, copper, and selenium also play critical roles in growth and development and should not be overlooked in the nutritional management of children with ASD. The only case reporting vitamin K and E deficiency was in conjunction with vitamin A as a secondary deficiency, and no clinical symptoms were reported [28]. Likewise, selenium and zinc were reported as secondary deficiencies in a 6-year-old with chronic restrictive eating, requiring enteral nutrition [25].

Furthermore, interactions between vitamins and minerals are also crucial, as they often work synergistically to support various physiological functions. For instance, erythropoiesis depends on a combination of iron, folate, vitamin B12, and vitamin A, vitamin D plays a key role in calcium metabolism, and vitamin C enhances the absorption of non-heme iron [1]. Approximately 70% of cases had two or more micronutrient deficiencies reported simultaneously (Figure 3). All cases of calcium deficiency were accompanied by vitamin D deficiency, 75% of iron-deficient cases had concurrent vitamin C deficiency, and 25% of vitamin A-deficient cases also had B12 deficiency (Table 1). The latter is unexpected as vitamin A (a fat-soluble vitamin) and vitamin B12 (a water-soluble vitamin) have distinctive absorption and metabolic pathways. However, both can be stored in the liver and play interconnected roles in physiological processes such as cell differentiation and erythropoiesis [1]. These findings provide valuable insight into the prevalence and clinical consequences of micronutrient deficiencies in children with ASD, highlighting the need for further exploration and clinical intervention.

## 4. Discussion

Micronutrient deficiencies are often considered rare in the general U.S. population due to widespread food fortification and nutritional supplementation [4]. While certain subpopulations, such as the elderly, pregnant women, and individuals with malabsorptive disorders, are well recognized as being at higher risk for micronutrient imbalances, there is comparatively less information available on high-risk pediatric populations, with the exception of vitamin C. However, this review found that nearly half (49.1%) of micronutrient deficiencies were due to fat-soluble vitamins such as vitamin D and A (Figure 2). Although the frequent testing for vitamin D may have influenced its prominence in the findings, this does not apply to vitamin A, which is tested less routinely. Similar trends have been observed in other countries. A case–control study among children with ASD in Qatar found that children with ASD had significantly lower serum vitamin D and iron levels compared to neurotypical controls, and authors concluded by suggesting the need for serum ferritin levels to be monitored in every case of ASD as part of baseline investigation not only hemoglobin [67]. The progression of deficiencies varies depending on the nutrients involved, and particularly solubility (fat or water soluble). For instance, rickets and xerophthalmia often follow a more protracted course compared to vitamin C deficiency, which can develop within eight weeks of inadequate intake [15]. This variability makes early identification of deficiencies challenging, as symptoms may not become apparent until deficiencies reach advanced stages. 

Moreover, the co-occurrence of multiple deficiencies raises concerns about the downstream effects of addressing a single deficiency only. Although treatment should prioritize the primary deficiency causing symptoms, addressing secondary or potential deficiencies must also be considered, as one nutrient deficiency can affect the bioavailability of another. Approximately 70% of cases reviewed reported two or more overlapping deficiencies (Figure 3), suggesting single-nutrient supplementation may not always be sufficient. For example, one case study documented vitamin A supplementation as a treatment of xerophthalmia without further assessment for other potential deficiencies, despite the presence of selective eating habits. Although the patient was eventually referred to a nutritionist, this was due to thin body habitus, as described by the authors [35]. Another case involved moderate iron deficiency alongside severe vitamin C deficiency; however, only vitamin C supplementation was provided, with no indication of iron supplementation or dietary interventions to improve iron status [29]. Case reports in Canada have also documented co-existing deficiencies of vitamin A, zinc, and vitamin D [52]; vitamin C, A, D, and zinc [68]; and vitamin C, iron, and vitamin D [69], with all reports emphasizing the importance of addressing secondary deficiencies as well.

Routine laboratory testing for micronutrient status in the pediatric population in the U.S. remains limited, with hemoglobin being one of the few regularly assessed markers for iron status [16]. In the absence of biochemical analysis, dietary assessment can provide critical insights into inadequate intake, while physical examinations may guide further investigation. However, symptom presentation can be inconsistent and variable among individuals. For example, pellagra caused by vitamin B3 deficiency typically presents as the triad of dermatitis, dementia, and diarrhea. However, in the two cases reviewed, the classic presentation was incomplete [22,36]. Dermatitis was the only reported symptom in both cases, and one patient was initially misdiagnosed with cellulitis and treated with antibiotics, leading to symptom worsening over time [36], thus the limitation of relying solely on physical examination, as micronutrient deficiencies can be missed without classic signs or biochemical confirmation, highlighting the need for dietary assessments during well-child visits to identify nutritional inadequacies early, beyond anthropometrics.

Although anthropometric measurements such as weight, height and Body Mass Index (BMI) are commonly used to assess nutritional status, our findings suggest that normal growth metrics do not rule out micronutrient deficiencies (Table 1). Previous studies by Yule et al. [14] and Sharp et al. [15] similarly reported that 62.9% and 67% of children with micronutrient deficiencies, respectively, had normal anthropometric measures according to the CDC growth chart. These findings align with the trends observed in this review; 50% of cases had a normal BMI, while the other half were identified as overweight or obese (Table 1). However, not all studies documented anthropometric data, limiting the ability to fully assess the relationship between growth metrics and micronutrient status in this population. Despite this, a consistent finding across all 44 cases was food selectivity, often characterized by restrictive dietary patterns (Table 1).

Feeding challenges in children with ASD are reported to be six times more common than in typically developing peers [70], with prevalence estimates ranging from 46% to 89% [7]. Across all 44 cases reviewed, dietary assessments consistently identified food selectivity, described using terms such as “picky eating”, “restricted food intake”, “selective eating”, and “limited diet” (Table 1). The lack of a standardized classification for these eating behaviors further complicates nutritional assessment. Studies indicate a preference for carbohydrate-rich foods, such as crackers, chips, and pasta, while rejecting fruits, vegetables, and protein-rich foods [8]. These preferences are have been associated with hypersensitivity or hyposensitivity to sensory stimuli, including textures, tastes, smells, and appearances [71]. Similarly, this review found commonly reported foods included crackers, potatoes (French fries or chips), pasta, and bread (Table 1). This pattern is not unique to the U.S., but has also been observed in other countries. Case reports and series in Ireland [55], Australia [56] and Japan [72] report that children with ASD consume lower amounts of protein, dairy, fruit and vegetables, and have stronger preference for processed and carbohydrate rich-foods. This further suggests that food selectivity in this population transcends cultural and regional differences requiring more attention.

Additionally, some children with ASD may meet criteria for avoidant/restrictive food intake disorder (ARFID), a recently recognized eating disorder in the DSM-5. ARFID is characterized by persistent food restriction not attributable to medical or cultural factors, sometimes leading to significant nutritional deficiencies, reliance on oral nutritional supplements, and, in some cases, the need for enteral feeding support [6]. Five cases in this review required enteral feeding—three with a nasogastric tube [34,44] and two gastrostomy tubes [25,28]. However, none of these cases explicitly reported ARFID diagnosis, suggesting that even without ARFID diagnosis, children with ASD remain at high risk for micronutrient deficiencies.

### 4.1. Limitations

The findings of this review should be interpreted within the context of some limitations. The inherent bias of case reports and case series limits the generalizability of findings to the broader population of children with ASD. The sample may not fully represent the spectrum of ASD severity, dietary patterns, or healthcare access. Additionally, since case reports often focus on individuals with more severe clinical presentations, milder or subclinical nutrient deficiencies may be underreported. Variability in the methods used to diagnose, document, and treat deficiencies further complicates data interpretation. While some cases included detailed biochemical analyses, dietary histories, and anthropometric data, others provided minimal or incomplete information. Furthermore, a dietary history was frequently obtained from caregivers and may have been subject to recall bias. Missing details regarding the duration of restrictive eating patterns, supplement use, prior medical interventions, and whether patients received nutrition counseling from a registered dietitian could impact the accuracy of reported deficiencies. Lastly, as all cases were authored by physicians, the information in case reports used may reflect a medical perspective, potentially overlooking nutritional insights that dietitians or other healthcare professionals might have emphasized.

### 4.2. Implications for Clinical Practice

Although these limitations raise concerns about generalizability, this review highlights the need for standardized procedures for assessing the nutritional status of children with ASD beyond anthropometric measures. Although routine micronutrient screening for all children with ASD based on reports of restrictive eating may be impractical due to healthcare costs and resource constraints, it is evident that children with ASD are at high risk for micronutrient deficiencies even when growth parameters are normal. Consequently, highlighting importance of involving nutrition professionals in patients’ care.

Registered dietitians were rarely mentioned in the initial evaluations of these cases, with most consulted after multivitamin supplementation was initiated, when feeding therapy was recommended or as a follow up service outpatient. It has been reported that most medical students receive fewer than 20 h of nutrition education during their training [73]; there is a critical need for enhanced nutrition education for physicians, improved collaboration between medical professionals, and increased referrals to nutrition services. Medical nutrition therapy provided by dietitians has been shown to improve clinical outcomes and reduce healthcare costs associated with malnutrition [74,75]. In the absence of clinical signs and symptoms, a comprehensive nutritional assessment by a registered dietitian may lead to early detection of deficiencies and targeted preventative interventions. Expanding access to nutrition care, particularly in outpatient settings, could help prevent hospitalizations due to nutritional deficiencies.

Notably, approximately 70% of cases reported two or more concurrent micronutrient deficiencies, raising the possibility that additional deficiencies may have gone undiagnosed in cases reporting only a single deficiency. Across all cases reviewed, treatment primarily involved micronutrient supplementation—administered orally, enterally, intravenously or intramuscularly—which resolved or improved symptoms, even in severe cases (Table 1). However, given the persistent restrictive dietary patterns observed in this population, future deficiencies are likely if replacement therapy is used only as a short-term intervention rather than as part of a comprehensive, individualized nutrition plan. Further research is needed to determine whether routine micronutrient testing, or supplementation should be recommended as a preventive measure in children with ASD at risk of nutritional deficiencies.

## 5. Conclusions

Children with autism spectrum disorder (ASD) are at high risk of micronutrient deficiencies, often resulting from restrictive eating patterns characterized by food selectivity and limited dietary variety. This review identified vitamin D, vitamin A, B-vitamins, calcium, and iron as the most frequently reported deficiencies, with clinical manifestations including xerophthalmia, rickets, pellagra, osteopenia, etc., and replacement therapy resolved most clinical symptoms. Despite the assumption that normal growth parameters indicate adequate nutritional status, deficiencies were present even in children with BMI within the normal range. Developing a standardized nutrition assessment tool or guideline for individuals with ASD may need further investigation due to limited research on large populations and inconsistencies in existing methodologies of cases reviewed. However, the findings of this review suggest that a combination of anthropometric, biochemical, and dietary assessment may serve as a foundation for future screening tools. Both clinical and anecdotal evidence highlight the need for a more proactive role by nutrition professionals to prioritize early nutritional intervention, preventing long-term complications and comorbidities.

## Figures and Tables

**Table 1:** Characteristics of cases of children 2–18 years with Autism Spectrum Disorder and micronutrient deficiency due to dietary pattern.

Reference, Year	Age and Sex	Anthropometrics	Dietary Pattern	Micronutrient Deficiency/Insufficiency	Deficiency Disease	Intervention	Outcome
Dixon (2024) [19]	10 y, female	Not reported	Self-restricted diet of almost exclusively fast food and French fries	Vitamin A	Xerophthalmia	Vitamin A supplementation	Supplementation resolved symptoms such as light sensitivity, epiphora, and redness in 3 weeks
Rittenhouse (2024) [20]	7 y, female	History of poor weight gain	Primarily consisted of cheese crackers and dry cereal	Vitamin C, vitamin D, iron	Scurvy	Oral vitamin C supplementation	Ambulatory function improved after a few days of supplementation, referral to orthopedics and feeding clinic
Marek (2023) [21]	7 y, male	Not reported	Mostly cheese quesadillas and chicken nuggets	Vitamin A	Xerophthalmia with keratomalacia	Oral vitamin A	Tear film and conjunctival appearance improved but vision did not improve (irreversible blindness)
Marek (2023) [21]	5 y, male	Not reported	Ate only “tan colored foods”	Vitamin A	Xerophthalmia	Intramuscular vitamin A	Right eye normalized but left eye had irreversible scarring (irreversible partial blindness)
Marek (2023) [21]	6 y, male	Not reported	Diet consisted of peanut butter, apple sauce and French fries	Vitamin A	Xerophthalmia	Oral vitamin A	Bilateral eye redness, tearing, and photophobia resolved with supplementation
Marek (2023) [21]	7 y, male	Not reported	Exclusively chicken fingers and French fries	Vitamin A, vitamin B12	Xerophthalmia, optic neuropathy	Vitamin A, D, E, K and B12 supplementation	Ocular surface and vision normalized
Marek (2023) [21]	14 y, male	Not reported	Chicken nuggets, rice, and pears	Vitamin A	Xerophthalmia	Oral vitamin A, D, E and K supplementation	Severe dry eyes, irritation, and photophobia resolved
Marek (2023) [21]	6 y, male	Not reported	Restricted diet due to sensory issues	Vitamin A	Xerophthalmia	Vitamin A supplementation	All signs and symptoms of xerophthalmia resolved
Griffin (2023) [22]	10 y, male	Not reported	Described as “picky eater”	Vitamin B3	Pellagra	Replacement therapy	Not reported
Hartman (2023) [23]	9 y, male	Not reported	Restricted diet of potatoes, bacon, and lettuce	Calcium, Vitamin D	Rickets, severe bone demineralization	Elemental calcium and ergocalciferol supplementation	Improved mobility and range of motion
Moore (2022) [24]	13 y, male	Weight and height within normal limits	Restrictive eating, accepted mostly bread and peanut butter from specific brands, chicken, no seafood, dairy or canned foods	Iodine, iron, vitamin C	Goiter, hypothyroidism	Iodine supplementation of 150 mcg/day, levothyroxine, multivitamin with iron	Significant improvement in size of goiter, TSH and free T4 normalized within one month
Quinn (2022) [25]	6 y, male	Not reported	Chronic restrictive eating limited initially to nutritional supplemental beverage and candy, but 6 months prior to encounter further restricted to diet to chocolate peanut butter cups and water	Vitamin C, vitamin D, vitamin A, iron, selenium, Vitamin B1, zinc	Scurvy	Vitamin C, thiamin, multivitamin within 24 h of admission, selenium supplementation started day 2. G-tube feeds, multivitamin, zinc and ferrous sulfate on discharge	Vitamin C, selenium, vitamin D and thiamine normalized on hospital day 9–14. At 3-month follow up, improved ambulatory function on left leg and no signs and symptoms of cardiac dysfunction
Ganta (2022) [26]	10 y, male	Not reported	Texture aversion, minimal dairy and protein intake	Vitamin D, calcium	Hypocalcemia	Intravenous calcium gluconate, oral calcium carbonate and cholecalciferol	Improved calcium, vitamin D and ambulatory function
Ganta (2022) [26]	14 y, male	Not reported	Pasta, potatoes, poultry	Vitamin D, calcium	Hypocalcemia	Oral calcium carbonate and cholecalciferol, surgery for displaced fracture	Improved calcium, vitamin D and ambulatory function
Sastry (2022) [27]	17 y, male (case 2)	Weight < 3%ile	Diet limited to potato chips, pretzels, waffles, and chocolate	Vitamin D, calcium	Severe vitamin D deficiency, hypocalcemia	Intravenous calcium gluconate, oral calcium carbonate, cholecalciferol and calcitriol for 8 days, then 4 cans of ensure per day, multivitamin, cholecalciferol upon discharge.	Normalized vitamin D and calcium 1-week post discharge
Godfrey (2022) [28]	17 y, male	BMI 94–95%ile ^†^	History of restrictive eating, prefers food rich in carbohydrates	Vitamin A, vitamin B12	Xeropthalmia, vision loss, nyctalopia, photosensitivity	Vitamin A supplementation and prednisone	Vision: initial improvement in night vision but vision worsened when prednisone was weaned
Godfrey (2022) [28]	17 y, male	BMI > 95%ile ^†^	Typically pre-packaged meals, heavy carbohydrate foods and soda intake	Vitamin A, vitamin E, vitamin K	Xeropthalmia, Vision loss, nyctalopia	Vitamin A, leucovorin, vitamin B1, vitamin D, vitamin K	Improved vision; however, not to baseline
Godfrey (2022) [28]	5 y, male	BMI 5–10%ile ^†^	Typically bread sticks, raw spinach, potato chips, waffle fries, and popcorn	Vitamin A, vitamin B12, vitamin E	Xeropthalmia, Vision loss, nyctalopia, photosensitivity	Vitamin A, vitamin E, vitamin B12 and g-tube placement	Improved vision; however, not to baseline
Godfrey (2022) [28]	16 y, male	BMI 25–50%ile ^†^	Restricted eating—mostly pre-packaged tortilla chips, salsa, and cheese puffs	Vitamin A	Xeropthalmia, nyctalopia, vision loss	Vitamin A	Slightly improved vision
Godfrey (2022) [28]	12 y, male	BMI > 95%ile ^†^	Long history of mild restrictive eating	Vitamin A, vitamin B12	Xeropthalmia, nyctalopia, vision loss	Vitamin A and B12 supplementation	Marginally improved vision
Godfrey (2022) [28]	9 y, male	BMI > 95%ile ^†^	History of restrictive eating, mostly bread products	Vitamin A	Xeropthalmia, Nyctalopia, vision loss	Vitamin A supplementation	Improved vision
Regehr (2021) [29]	18 y, male	Not reported	Severe food aversion, diet consisted of exclusively pop-tarts	Vitamin C, iron, vitamin D	Scurvy	Vitamin C 1000 mg/day, discharged on oral vitamin C 250 mg/day and vitamin D 1000 IU/day	Improved ambulatory function, less joint swelling, no further gum bleeding
Hartman (2021) [30]	11 y, female	BMI 25–50%ile ^†^	Described as a “picky eater” diet consisted of French fries, chicken nuggets, waffles, unfortified orange juice, yellow rice, and some meat and fish	Vitamin D, calcium	Rickets, hypocalcemia	Intravenous calcium gluconate, oral calcium carbonate, calcitriol and ergocalciferol, orthopedic surgery for genu valgum (knock knees)	Improved calcium, vitamin D, and ambulatory function
Jacob (2021) [31]	13 y, male	BMI 85%ile–94%ile	Described as “picky eater” diet consisted of mostly potato chips, French fries, corn, crackers, pizza without cheese, chicken nuggets, ginger ale, and apple juice	Vitamin D, calcium, vitamin A, vitamin B12, vitamin B6 and zinc	Severe vitamin D deficiency, hypocalcemia, diffuse osteopenia, femoral metaphyseal fractures	Intravenous calcium gluconate 2 g every 2–3 h for 2 days, bilateral antegrade nailing of femurs, vitamin D3 50,000 IU 1x/week. Postoperatively, 120 mg/kg/day of calcium carbonate every 8 h	Improved calcium and vitamin D, discharged to rehab for feeding and physical therapy
Raouf (2021) [32]	15 y, male	Not reported	Restricted to intake of bread, pasta, rice, and potatoes	Vitamin A, vitamin D	Bilateral progressive blurry vision, nyctalopia	Vitamin A supplementation and surgical optic nerve decompression	Improvement in visual acuity of both eyes
Luckow (2020) [33]	5 y, male	Not reported	Restricted diet, preferences for carbohydrate foods	Vitamin C, Vitamin D	Scurvy, gingivitis	Vitamin C, vitamin D and multivitamin supplementation	Not reported but discharged with plan to follow up in nutrition clinic
Fortenberry (2020) [34]	7 y, male	Weight 25 kg	Diet limited to nacho cheese flavored chips, cheddar flavored crackers, and apples	Vitamin D, vitamin B12, vitamin C	Scurvy	Intravenous multivitamin	On discharge vitamin C, bleeding, pain and range of motion of lower extremities improved
Fortenberry (2020) [34]	10 y, male	Weight 32 kg	Mostly fast-food quesadillas, cheeseburgers, French fries, macaroni and cheese, peanut butter sandwiches, and pizza	Iron, vitamin C	Scurvy	5 mL/kg of packed red blood cell, 250 mg/day vitamin C and liquid multivitamin	Improved swelling along calves
Fortenberry (2020) [34]	10 y, male	Weight 38 kg	Limited diet of mostly chicken nuggets and chocolate milk	Iron, vitamin C	Scurvy	Elemental iron 65 mg 2x/day and ascorbic acid 125 mg 2x/day	During follow up bleeding and pain was reported to have improved
Fortenberry (2020) [34]	6 y, male	Weight 25 kg	Limited diet of mostly peanut butter candies, fast food cheeseburger, grilled cheese, chips and soda	Vitamin D, vitamin C	Scurvy	Ergocalciferol 50,000 IU/week, ascorbic acid 250 mg/day, elemental iron 24 mg 3x/day, multivitamin daily and nasogastric tube for nutrition support	Improved leg pain and gum bleeding, lost to follow up
Fortenberry (2020) [34]	14 y, male	Weight 31 kg	Mostly French fries, lemon-lime soda, chocolate bars	Vitamin C, folate, vitamin D, vitamin A, vitamin B1, vitamin B6	Scurvy, diffuse osteopenia	IV multivitamin, vitamin C 200 mg IV and ergocalciferol 50,000 units, enteral nutrition (nasogastric tube)	Improved ambulatory symptoms and lower extremity pain but discharged with wheelchair and rolling walker
Chan (2020) [35]	7 y, male	Thin body habitus	Selective diet of dry cereal, soda, juice	Vitamin A	Keratomalacia, photophobia	Single intramuscular dose of vitamin A, daily oral multivitamin supplement	Resolved bilateral xerophthalmia at one month follow up but corneal scars remained
Zaenglein (2020) [36]	10 y, male	BMI 50%ile–75%ile ^†^	Long term pattern of selective eating, consisting of mainly apples, popcorn, potato chips, cheese puffs and chocolate milk	Vitamin B3	Pellagra, photosensitive dermatitis	Niacinamide 50 mg 3x/day, daily multivitamin and feeding therapy	Skin improved within one week of supplementation
Perkins (2020) [37]	16 y, Male (case 4)	BMI 90%ile–95%ile ^†^	Crackers, oatmeal, peanut butter and jelly	Vitamin D, vitamin C	Scurvy	Vitamin D and C supplementation	Symptoms such as petechiae, gingivitis, joint swelling and pain improved
Perkins (2020) [37]	5 y, male (case 7)	BMI > 95%ile ^†^	Mostly orange yogurt, rice, black beans	Vitamin D, vitamin C	Scurvy	Vitamin D and C supplementation	Symptoms such as petechiae, gingivitis, joint swelling and pain improved
Perkins (2020) [37]	12 y, Male (case 8)	BMI 10–25%ile ^†^	Mostly ate graham crackers, cheese crackers and French fries	Vitamin D, vitamin B12, vitamin C	Scurvy	Vitamin D, B12 and C supplementation	Symptoms such as petechiae and gingivitis improved
Stalnaker (2019) [38]	3 y, male	Not reported	Extreme picky eating, only accepts crackers, potatoes and juice	Vitamin D, calcium	Hypocalcemic rickets	Vitamin D and calcium supplementation	Improved ambulatory and ASD symptoms one month after discharge
Shah (2019) [39]	5 y, male	BMI 85%ile	History of food selectivity to cookies, French fries	Vitamin D, calcium	Rickets, hypocalcemia	Intravenous calcium for a few days, followed by oral vitamin D3 50,000 IU and oral calcium 1600 mg/day	Resolution of electrocardiographic changes and normalized serum calcium and vitamin D
Burd (2019) [40]	4 y, female	BMI 25%ile	Limited and unpredictable diet with food aversion-beans, rice and cereal mostly	Low hemoglobin, hematocrit, iron, iron saturation	Iron deficiency anemia	Ferrous sulfate 44 mg/5 mL 2.5 mL 2x/day (reported patient sometimes refused it), iron rich foods plus orange juice	Mild change in iron labs such as hemoglobin and ferritin in one month
Tripathi (2018) [41]	9 y, female	BMI 12%ile	Picky eater—rice, fries, potato chips, homemade green juice/smoothies	Vitamin D, calcium	Rickets	Calcium carbonate, calcitriol and vitamin D3 supplementation	Significant improvement of pain and gait in about one month
Booms (2016) [42]	5 y, male (case 1)	Not reported	Limited diet to organic, gluten and dairy free foods, and non-iodized salt	Iodine	Goiter	Iodine supplementation 160 mcg/day and levothyroxine	Improved thyroid function and goiter resolved
Meisel (2015) [43]	3 y, male (case 1)	Not reported	Selective diet lacking fruits and vegetables	Vitamin C, vitamin A	Scurvy	Vitamin C and A replacement	Gingival hyperplasia, bleeding and rash rapidly improved
Baird (2015) [44]	11 y, male	Weight 71%ile	Restricted diet, only accepted chicken nuggets and occasional French fries	Vitamin A, B6, B1, copper, iron	Bilateral optic neuropathy	Parenteral pyridoxine 100 mg/day, thiamin 25 mg/day, multivitamin, and enteral nutrition (nasogastric tube)	Lactic acidosis, mental status and seizures improved with supplementation
Kitcharoensakkul (2014) [45]	5 y, male (case 3)	Weight and height < 3%ile	Restricted diet consisting of taquitos, frozen pizza rolls, crust of fish sticks and nacho cheese snacks	Vitamin C, vitamin D	Scurvy, osteopenia	Vitamin D and vitamin C replacement	Improved ambulatory function with assistance 2 weeks after discharge

BMI—Body Mass Index, kg—kilogram, mL—milliliters, mg—milligrams, mcg—micrograms, IU—international units, TSH—thyroid stimulating hormone, T4—thyroxine, %ile—percentile. Anthropometric status per the CDC growth chart 2–20 years old: underweight—BMI < 5th percentile, normal weight—5th to 84th percentile, overweight—BMI ≥ 85th and ≤ 95th percentile, obesity—BMI ≥ 95th percentile. Percentiles are used to assess children’s growth in the U.S.; thus, BMI in kg/m^2^ was converted using the CDC growth chart 2–20 years, indicated with ^†^.

## Supplementary Materials

The following supporting information can be downloaded at https://www.mdpi.com/article/10.3390/nu17060990/s1. Table S1: Reported Micronutrient Deficiency/Insufficiency Levels in Children with Autism Spectrum Disorder from case reports and series
