# Supplementary material for: The Increasing Prevalence of Autism Spectrum Disorder in the U.S. and Its Implications for Pediatric Micronutrient Status: A Narrative Review of Case Reports and Series
Source: Nutrients. 2025 Mar 12;17(6):990. doi: 10.3390/nu17060990 (PMC11945165; doi:10.3390/nu17060990)
Supplement: Supplementary file 1 [file nutrients-17-00990-s001.zip › nutrients-3509572-supplementary.pdf]

Table S1: Reported Micronutrient Deficiency/Insufficiency Levels in Children with Autism Spectrum Disorder from case reports and series				
**Reference, year**	**Age & Sex**	**Micronutrient Deficient/Insufficient**	**Vitamin/Mineral Level [Reference range]**	**Nutritional Deficiency Disease**
**Dixon (2024)[19]**	10 y, female	Vitamin A	Not reported but documented as severe	Xerophthalmia
**Rittenhouse (2024)[20]**	7 y, female	Vitamin C, vitamin D, iron	Vitamin C <5 µmol/L [43.6- 108.0]- severe<br>Vitamin D 24 ng/mL [30-100]- moderate<br>Iron 21 µg/dL [37- 145]- moderate	Scurvy
**Marek (2023)[21]**	7 y, male	Vitamin A	<0.06 mg/L [0.20–0.50]- severe	Xerophthalmia with keratomalacia
**Marek (2023)[21]**	5 y, male	Vitamin A	<0.06 mg/L [0.20–0.50]- severe	Xerophthalmia
**Marek (2023)[21]**	6 y, male	Vitamin A	<0.06 mg/L [0.20–0.50]- severe	Xerophthalmia
**Marek (2023)[21]**	7 y, male	Vitamin A, vitamin B12	Vitamin A <0.06 mg/L [0.20–0.50]- severe	Xerophthalmia, optic neuropathy

			Vitamin B12 226 pg/mL [271–1170]- moderate	
**Marek (2023)[21]**	14 y, male	Vitamin A	<0.06 mg/L [0.20–0.50]- severe	Xerophthalmia
**Marek (2023)[21]**	6 y, male	Vitamin A	<0.06 mg/L [0.20–0.50]- severe	Xerophthalmia
**Griffin (2023)[22]**	10 y, male	Vitamin B3	Not reported but documented as severe	Pellagra
**Hartman (2023)[23]**	9 y, male	Calcium, Vitamin D	Calcium 5.7 mg/dL [8.5–10.1]- moderate<br>Vitamin D < 4.0 ng/mL [30-100 ng/mL]- severe	Rickets, severe bone demineralization
**Moore (2022)[24]**	13 y, male	Iodine, iron, vitamin C	Iodine <5.0 µg/L [26-705 µg/L]- severe<br>TIBC 505 µg/dL [Not reported]- documented as mild<br>Vitamin C <5 µmol/L [Not reported]- documented as severe	Goiter, hypothyroidism

**Quinn (2022)[25]**	6 y, male	Vitamin C, vitamin D, vitamin A, iron, selenium, Vitamin B1, zinc	Vitamin C <0.1 mg/dl [0.4-2.0]- severe<br>Vitamin D <3.5 ng/ml [30-96]- severe<br>Vitamin A 15 µg/dl [19-77]- mild<br>Iron saturation 12% [20-55]- moderate<br>Ferritin 16.6 ng/ml [≥ 20]- mild<br>Selenium 43 ng/ml [70-150]- moderate<br>Vitamin B1 55 nmol/L [70-180]- moderate<br>Zinc 0.39 µg/ml [0.6- 1.2]- mild	Scurvy
**Ganta (2022)[26]**	10 y, male	Vitamin D, calcium	Vitamin D 8.2 ng/mL [30-100]- severe<br>Calcium 4.8 mg/dL [8.5–10.5]- moderate	Hypocalcemia
**Ganta (2022)[26]**	14 y, male	Vitamin D, calcium	Vitamin D 9.4 ng/mL [30-100]- severe<br>Calcium 5.5 mg/dL [8.5–10.5]- moderate	Hypocalcemia
**Sastry (2022)[27]**	17 y, male (case 2)	Vitamin D, calcium	Vitamin D <4 ng/mL [25-100]- severe<br>Calcium <5 mg/dL [8.8–10.8]- moderate	Severe vitamin D deficiency, hypocalcemia
**Godfrey (2022)[28]**	17 y, male	Vitamin A, vitamin B12	Vitamin A 5.6 µg/dL [Not reported]- documented as severe	Xeropthalmia, vision loss, nyctalopia,

			Vitamin B12 298 µg/dL [Not reported]-documented as low	photosensitivity
**Godfrey (2022)[28]**	17 y, male	Vitamin A, vitamin E, vitamin K	Vitamin A <10 µg/dL [Not reported]-documented as severe<br>Vitamin E 5.0 µg/dL [Not reported]-documented as low<br>Vitamin K 0.2 µg/dL [Not reported]-documented as low	Xeropthalmia, Vision loss, nyctalopia
**Godfrey (2022)[28]**	5 y, male	Vitamin A, vitamin B12, vitamin E	Vitamin A <2.0 µg/dL [Not reported]-documented as severe<br>Vitamin E 6.8 µg/dL [Not reported]-documented as low<br>Vitamin B12 243 µg/dL [Not reported]-documented as low	Xeropthalmia, Vision loss, nyctalopia, photosensitivity
**Godfrey (2022)[28]**	16 y, male	Vitamin A	Vitamin A 4.4 µg/dL [Not reported]-documented as severe	Xeropthalmia, nyctalopia, vision loss

**Godfrey (2022)[28]**	12 y, male	Vitamin A, vitamin B12	Vitamin A <2 μg/dL [Not reported]- documented as severe<br>Vitamin B12 431 μg/dL [Not reported]- documented as low	Xeropthalmia, nyctalopia, vision loss
**Godfrey (2022)[28]**	9 y, male	Vitamin A	Vitamin A 3.8 μg/dL [Not reported]- documented as severe	Xeropthalmia, Nyctalopia, vision loss
**Regehr (2021)[29]**	18 y, male	Vitamin C, iron, vitamin D	Vitamin C <0.01 mg/dL [0.6-2.0]- severe<br>Vitamin D <3 ng/mL [20-50]- severe	Scurvy
**Hartman (2021)[30]**	11 y, female	Vitamin D, calcium	Vitamin D <10 ng/mL [> 30]- severe<br>Calcium 5.3 mg/dL [8.5–10.5]- moderate	Rickets, hypocalcemia
**Jacob (2021)[31]**	13 y, male	Vitamin D, calcium, vitamin A, vitamin B12, vitamin B6 and zinc	Vitamin D <4 ng/ml [30-80]- severe<br>Calcium 4.39 mg/dL [8.4-10.2]- moderate<br>Vitamin A 0.13 mg/Dl [0.26-0.70]- moderate<br>Vitamin B12 124 pg/mL [211-911]- moderate<br>Vitamin B6 8.6 mmol/L [20-125]- moderate<br>Zinc 31.2 μg/dL [60-120]- moderate	Severe vitamin D deficiency, hypocalcemia, diffuse osteopenia, femoral metaphyseal fractures

**Raouf (2021)[32]**	15 y, male	Vitamin A, vitamin D	Vitamin A undetectable [18.8- 54.9]- severe<br>Vitamin D 19.8 ng/mL [30-100]- moderate	Bilateral progressive blurry vision, nyctalopia
**Luckow (2020)[33]**	5 y, male	Vitamin C, Vitamin D	Vitamin C <0.1 mg/dL [0.4-20]- severe<br>Vitamin D 19 ng/mL [30-100]- moderate	Scurvy, gingivitis
**Fortenberry (2020)[34]**	7 y, male	Vitamin D, vitamin B12, vitamin C	Vitamin D 11 ng/ml [>19]- moderate<br>Vitamin B12 183 pg/mL [210- 1033]- moderate<br>Vitamin C < 0.1 mg/dL [0.4-2.0]- severe	Scurvy
**Fortenberry (2020)[34]**	10 y, male	Iron, vitamin C	Vitamin C < 0.1 mg/dL [0.4-2.0]- severe<br>Iron 15 µg/dL [50-120]- moderate	Scurvy
**Fortenberry (2020)[34]**	10 y, male	Iron, vitamin C	Vitamin C < 0.1 mg/dL [0.4-2.0]- severe<br>Iron 11 µg/dL [50-120]- moderate	Scurvy
**Fortenberry (2020)[34]**	6 y, male	Vitamin D, vitamin C	Vitamin D 14.3 ng/ml [>19]- mild<br>Vitamin C < 0.1 mg/dL [0.4-2.0]- severe	Scurvy

**Fortenberry (2020)[34]**	14 y, male	Vitamin C, folate, vitamin D, vitamin A, vitamin B1, vitamin B6	Vitamin C 0.15 mg/dL [0.4-2.0]- severe<br>Folate 3.9 ng/mL [> 4.8]- mild<br>Vitamin D 7.2 ng/ml [>19]- moderate<br>Vitamin A 1.4 mg/dL [3-6]- moderate<br>Vitamin B1 38 nmol/L [70-180]- moderate<br>Vitamin B6 12.4 nmol/L [20.125]- moderate	Scurvy, diffuse osteopenia
**Chan (2020)[35]**	7 y, male	Vitamin A	Vitamin A undetectable [17- 56 µd/dL]- severe	Keratomalacia, photophobia
**Zaenglein (2020)[36]**	10 y, male	Vitamin B3	Nicotinamide & nicotinic <20ng/ml [Not reported]- documented as severe	Pellagra, photosensitive dermatitis
**Perkins (2020)[37]**	16 y, Male (case 4)	Vitamin D, vitamin C	Vitamin D not reported<br>Vitamin C <0.1 mg/dL [0.4-2.0]- severe	Scurvy
**Perkins (2020)[37]**	5 y, male (case 7)	Vitamin D, vitamin C	Vitamin D not reported<br>Vitamin C <0.1 mg/dL [0.4-2.0]- severe	Scurvy
**Perkins (2020)[37]**	12 y, Male (case 8)	Vitamin D, vitamin B12, vitamin C	Vitamin D not reported, documented as low	Scurvy

			Vitamin B12 not reported, documented as low<br>Vitamin C <0.1 mg/dL [0.4-2.0]- severe	
**Stalnaker (2019)[38]**	3 y, male	Vitamin D, calcium	Vitamin D 7.6 ng/mL [20–100]- moderate<br>Calcium 6.0 mg/dL [Not reported]	Hypocalcemic rickets
**Shah (2019)[39]**	5 y, male	Vitamin D, calcium	Vitamin D <5 ng/mL [30-100]- severe<br>Calcium 4.4 mg/dL [9.2–11.0]- moderate	Rickets, hypocalcemia
**Burd (2019)[40]**	4 y, female	Low hemoglobin, hematocrit, iron, iron saturation	Hemoglobin 10.6 g/dL [12- 15]- mild<br>Hematocrit 31.9% [37-43]- mild<br>Iron 30 µg/dl [50-170]- moderate<br>Iron saturation 9% [10-50]- mild	Iron deficiency anemia
**Tripathi (2018)[41]**	9 y, female	Vitamin D, calcium	Vitamin D 2.8 ng/mL [30-100]- severe<br>Calcium 6.4 mg/dL [9.1-10.5]- moderate	Rickets
**Booms (2016)[42]**	5 y, male (case 1)	Iodine	Iodine 11 µg/L UIC [<20- 99]- moderate<br>Free T4 0.18 ng/dL [0.84–2.26]- moderate	Goiter

**Meisel (2015)[43]**	3 y, male (case 1)	Vitamin C, vitamin A	Vitamin C <0.1 mg/dL [0.6- 2]- severe<br>Vitamin A 9.2 mg/dL [11.3-64.7]- moderate	Scurvy
**Baird (2015)[44]**	11 y, male	Vitamin A, B6, B1, copper, iron	Vitamin A <0.06 mg/L [0.2-0.5]- severe<br>Vitamin B6 7.6 ng/mL [20-125]- moderate<br>Iron 32 µg/dL [41-141]- mild<br>Copper 55 µg/dL [90-120]- moderate	Bilateral optic neuropathy
**Kitcharoensakkul (2014)[45]**	5 y, male (case 3)	Vitamin C, vitamin D	Vitamin D 16 ng/mL [30-100]- moderate<br>Vitamin C <0.1 mg/dL [0.6- 2.0]- severe	Scurvy, osteopenia

µmol/L= micromoles per liter
ng/mL= nanograms per milliliter
µg/dL= micrograms per deciliter
mg/L= milligrams per liter
pg/mL= picograms per milliliter
mg/dL= milligrams per deciliter
ng/ml= nanograms per milliliter
nmol/L= nanomoles per liter
UIC= urinary iodine concentration
Note: Some case reports and series did not specify the exact levels of vitamin deficiencies, instead describing them as "low." Additionally, some reports that provided deficiency levels did not include the reference ranges used for comparison.
